# Case Report: Oral and fecal microbiota in a super-donor: the healthy microbiota paradigm for fecal transplantation

**DOI:** 10.3389/frmbi.2023.1219960

**Published:** 2023-09-12

**Authors:** Nayeli Ortiz-Olvera, Edith A. Fernández-Figueroa, Jesús Argueta-Donohué, Haydee Miranda-Ortíz, Erika Ruiz-García

**Affiliations:** ^1^ Departamento de Gastroenterología, UMAE, Hospital de Especialidades, Centro Médico Nacional Siglo XXI, IMSS, Ciudad de Mexico, Mexico; ^2^ Núcleo B de Innovación en Medicina de Precisión. Instituto Nacional de Medicina Genómica, Ciudad de México, Mexico; ^3^ Laboratorio de Neurofarmacología, Instituto Nacional de Psiquiatría Ramón de la Fuente Muñiz, Ciudad de México, Mexico; ^4^ Unidad de Secuenciación, Instituto Nacional de Medicina Genómica, Ciudad de México, Mexico; ^5^ Departamento de Tumores de Tubo Digestivo, Instituto Nacional de Cancerología, Ciudad de México, Mexico; ^6^ Laboratorio de Medicina Traslacional, Instituto Nacional de Cancerología, Ciudad de México, Mexico

**Keywords:** oral microbiota, fecal microbiota, *F. prausnitzii*, *F. nucleatum*, *C. difficile*

## Abstract

Despite the numerous fecal microbiota transplantation trials that have been carried out, knowledge about the actual composition of a “healthy microbiota” remains scarce. The aim of this research was to describe the differences in the composition of oral and fecal microbiotas in a super-donor. The microbiota analysis was done using next-generation sequencing of the V3 and V4 regions of the bacterial 16S rRNA gene. The biodiversity found in the mouth was very rich, with 56 species identified, and there was a predominance of the genera *Veillonella*, *Haemophilus*, and *Streptococcus*. It is worth mentioning the presence (2.33%) of *Fusobacterium nucleatum* in the mouth. In feces, the genera *Bacteroides* and *Faecalibacterium* predominated, with the species *F. prausnitzii* being the most abundant. This analysis shows that the diversity of the microbiota of a super-donor plays a fundamental role in the effectiveness of its product in fecal matter transplantation. This suggests that transplanted gut microorganisms have the ability to maintain or recover health in a dynamic process between the microbiota and the host. Our findings encourage further research which will result in the development of bacterial therapies in infectious and inflammatory diseases.

## Introduction

Despite the numerous fecal microbiome transplantation (FMT) trials that have been carried out, knowledge about the actual composition of a “healthy microbiota” remains scarce. We do not know exactly how transplanted bacteria survive, colonize, and function in the recipient’s gut or, more importantly, what methods can be used to monitor transplanted feces, how to assess whether the product is suitable for transplantation, and whether it contains a “healthy microbiota” (Ianiro et al., 2014; Mukhopadhya et al., 2019).

The availability of novel tools such as next-generation sequencing (NGS) has allowed the identification of genes, and even complete genomes, that have been recovered from complex microbial communities (Human Microbiome Project Consortium, 2012; D’Amore et al., 2016).

Research has shown that some individuals have gut microbiomes with specific characteristics, such as the presence or absence of specific bacteria, phages, and metabolites (unfortunately not fully recognized), which protect the individual donor from the vast majority of gut dysbiosis (Wilson et al., 2019). These individuals are ideal donors and have been called “super-donors” because their fecal microbiota is very suitable for transplantation. However, recent studies have shown that there are some specific characteristics that make the microbiota of super-donors not universally suitable for transplantation (Olesen and Gerardin, 2021).

In this study, the variable regions V3 and V4 of the ribosomal 16S rRNA gene were amplified and sequenced to characterize the oral and fecal microbiota of a “super-donor” whose sample was used in 11 fecal microbiota recipients with *Clostridioides difficile* infection (8 recurrent and three severe) and who presented a favorable response. All patients presented with remission of diarrhea for a single FMT, motivating us to learn more about the bacterial composition of this “super-donor.”

## Materials and methods

### Donor and preparation of fecal microbiota

A single super-donor was recruited and screened following international (American and European) guidelines for fecal microbiota donors (Cammarota et al., 2019). The donor was interviewed about her medical history and lifestyle. Before analyzed the samples, were excluded a history of exposure to infectious agents, drug abuse, and risky social or sexual behavior in the last 6 months.in the donnor. The Rome IV questionnaire was applied to rule out any gut–brain interaction disorder, organic gastrointestinal diseases (celiac disease, inflammatory bowel disease, cancer), metabolic diseases (diabetes mellitus, thyroid pathology, hyperlipidemia), and neurologic diseases. In addition, the super-donor underwent a complete physical examination and laboratory tests. The latter included a complete blood count, and tests for glycemia, electrolytes, inflammatory markers, liver function, and thyroid function. In addition, screening serologies were performed for human immunodeficiency virus (HIV), syphilis, cytomegalovirus, herpes zoster, and hepatitis A, B, and C viruses.

A microbiological stool analysis was performed to rule out pathogenic bacteria (*Shigella* spp., *Salmonella* spp., *Campylobacter* spp., *Yersinia* spp., and *C. difficile*); as well as a stool antigen test to rule out *Helicobacter pylori*. All test results were negative. In addition, no rotavirus or parasite eggs were found in the feces.

The super-donor was a healthy, 26-year-old Mexican woman who was a non-smoker, did not take any medication, and had a body mass index (BMI) of 24.7 kg/m^2^. From the frequency of food intake, we could see that the average energy intake in 24 h was 1649.8 kcal, which was broken down into carbohydrates 221.3 g, protein 62.8 g, fat 57.6 g, saturated fat 18.72 g, fiber 23.2 g, vitamin A 694.2 mg, vitamin C 156.7 mg, calcium 620.3 mg, iron 9.9 mg, and sodium 2752.5 mg. Energy expenditure in 24 h was calculated at 1576.2 kcal.

### Sampling

A 4-hour fasting oral scraping was performed with a swab; we tried to take a homogeneous sample from the entire mouth. The sample was placed in 1 mL of nuclear lysis solution. In addition, the donor collected the first bowel movement of the day. Samples were frozen until analysis.

### Extraction of DNA and libraries

DNA extraction from the oral scraping was performed using the Wizard® Genomic DNA Purification Kit (cat. no. A1120; Promega), and DNA from the fecal sample was extracted at room temperature using the QIAamp® PowerFecal® Pro DNA Kit (cat. 51804; QIAGEN®), following the manufacturer’s instructions. Both the DNA samples were quantified and adjusted to 25 ng/μL. The integrity of the DNA was verified in a 1% agarose gel stained with SYBR™ Gold Nucleic Acid Gel Stain (cat. no. S11494; Invitrogen). To create the libraries, 250 ng of DNA was used and the primers reported in the Illumina 16S Metagenomic Sequencing Library Preparation protocol were used to amplify the V3 and V4 regions of bacterial 16S.

**Table:** 

Forward primer (3′–5′)	TCGTCGGCAGCGTCAGATGTGTATAAGAGACA GCCTACGGGNGGCWGCAG
Reverse primer (3′–5′)	GTCTCGTGGGCTCGGAGATGTGTATAAGAGACA GGACTACHVGGGTATCTAATCC

The fragments were amplified using *Taq* PCR Master mix 2× (cat. no. 1007544; QIAGEN), 1 μL (10 μM) of each primer, DNase- and RNase-free water in a final volume of 50 μL. The PCR assay was as follows: an initial denaturation at 94°C for 3 min, followed by 35 amplification cycles at 94°C for 40 s, 55°C for 2 min, and 72°C for 1 min, and a final extension at 72°C for 10 min. The PCR products were separated on a 1.5% agarose at 100 V for 1 h; the gels were stained as was described. The PCR products were purified using 75 μL of the Agencourt AMPure XP Kit (cat. no A63882; Beckman Coulter™) and eluted in RNase-free water.

### Sequencing and analysis

Both the samples were sequenced with an Illumina MiSeq platform, 2  ×  250, 500 cycles (Illumina Inc.). The reads were processed using DADA2 v1.12 to obtain error-corrected amplicon sequence variant representatives analogous to operational taxonomic units with single-nucleotide resolution (sOTUs). sOTUs annotated as chloroplasts and mitochondria were removed from the analysis.

The sOTUs were annotated with genus-level taxonomy using the Ribosomal Database Project (RDP)-naive Bayesian classifier, as implemented in DADA2, and, if possible, to species level using DADA2, both against the SILVA 16S database, v132.

The resulting OTUs were filtered for possible non-specific amplification using SortMeRNA v2.0 and Infernal v1.1.2. sOTUs with less than one read across all samples were excluded. Multiple sequence alignments of the sOTUs were performed using Infernal v1.1.2. A generalized linear mixed model (GLMM) was calculated for each sOTU to estimate its abundance (i.e., read counts) in relation to predictors. The package glmmTMB v0.1.4 was used to estimate the abundance of each microbe under a zero-inflated Poisson distribution. A multiple hypothesis correction was calculated using the Benjamini–Hochberg test.

### Plots

The alluvial plots were done in R software (The R Foundation for Statistical Computing, Vienna, Austria) version 4.4.2 and the R packages tidyverse and ggalluvial version 1.3.2 and 0.12.3, respectively, were used. Venn diagrams were created using Microsoft Excel® (Microsoft Corporation, Redmond, WA, USA).

## Results

### Determining oral and fecal biodiversity

**Figure 1:**
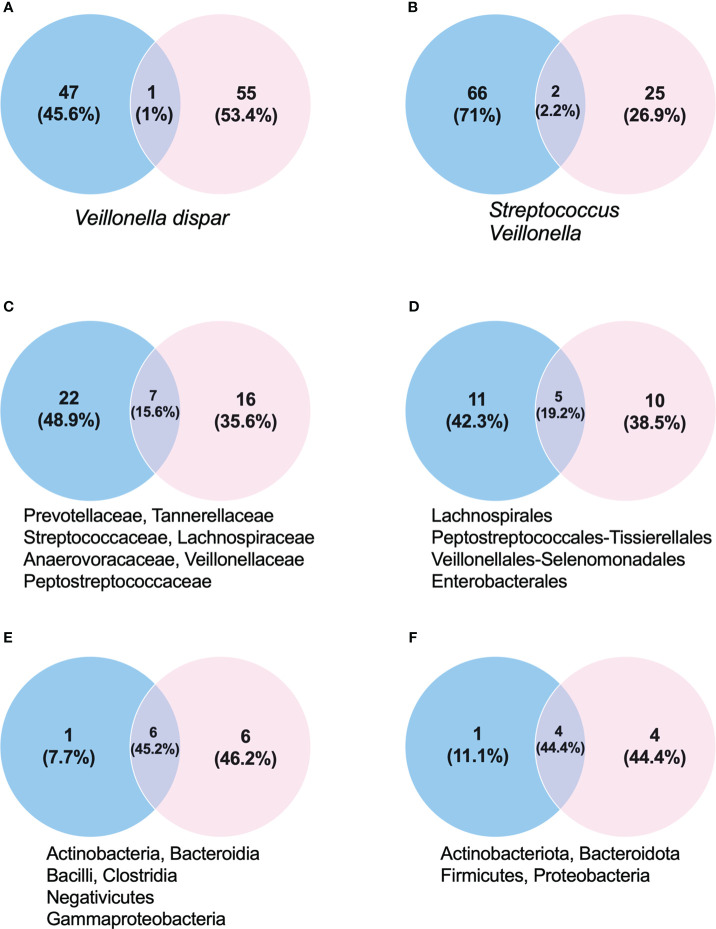
Proportion of taxonomic groups in stool (blue) and mouth (pink) samples. **(A)** Species. **(B)** Genus. **(C)** Families. **(D)** Order. **(E)** Class. **(F)** Phylum. Below each Venn diagram there are the taxonomic classification names shared between the oral and fecal samples.

A total of 14,696 and 39,303 sequencing readings of oral and fecal samples were obtained, respectively. The assignment of OTUs for the oral and fecal samples are shown in **Table 1** and **Figure 1**. The general classification of the mouth sample showed that 90.5% of all readings were bacterial, none were Archaea, and 9.5% were not identified at the genus level. The general classification of the fecal sample showed that 92% of all readings were bacterial, none were Archaea, and 8% were not identified at the genus level.

**Table 1:** Number of OTUs identified in stool and oral cavity in the super-donor.

Sample	Phylum	Class	Order	Family	Genus	Species
**Stool**	5	7	16	31	69	48
**Oral**	8	12	15	23	28	56

OTUs, Operational Taxonomic Units.

### Oral microbiota

**Figure 2:**
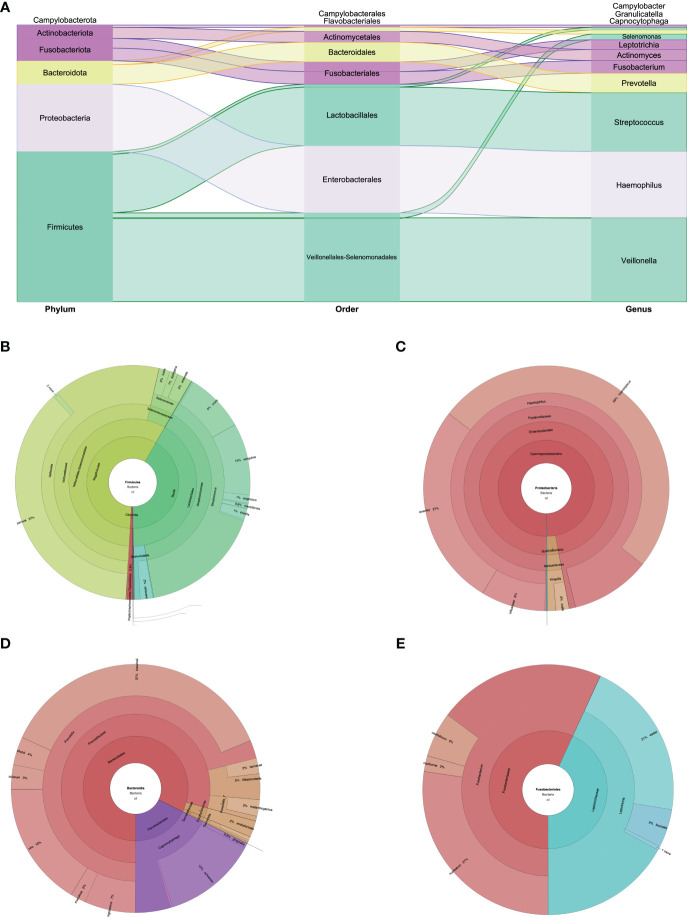
**(A)** Phylum, order, and genus enrichment in the oral cavity sample of the super-donor. Firmicutes (green), Proteobacteria (lilac), Bacteroidota (yellow), Fusobacteria and Actinobacteria (purple), and Campylobacterota (without color) were the most represented phyla in the oral cavity sample. **(B)** Taxonomic classification of the Firmicutes phylum. **(C)** Taxonomic classification of the Proteobacteria phylum. **(D)** Taxonomic classification of the Bacteroidota phylum. **(E)** Taxonomic classification of the Fusobacteriota phylum.

The biodiversity found in the mouth was very rich, with 56 species identified. Of the eight phyla found, Firmicutes, Proteobacteria, and Bacteroidota predominated. Fusobacteriota, Actinobacteria, and Campylobacterota were also found, but in lower percentages (**Figures 1**, **2**). In the Firmicutes phylum, the predominant class was Negativicutes of the Veillonellales–Selenomonadales order, with a clear predominance of *Veillonella parvula* at 19.7%; in the Bacilli class, Lactobacillales order of the Streptococcus family, the predominant species were *Streptococcus sanguinis* at 5.25% and *Streptococcus oralis* at 4.32% (**Figures 1**, **2A, B**). In the Proteobacteria phylum, the Gammaproteobacteria class, Pasteurellaceae family, *Haemophilus* genus, *Haemophilus haemolyticus* species predominated at 11.54%, followed by *Haemophilus quentini* at 6.14% (**Figures 1**, **2A, C**). On the other hand, in the Bacteroidales order, family Prevotellaceae, the *Prevotella* genus prevailed with respect to the Bacteroidota phylum, and the species with greater abundance was *Prevotella loescheii* at 3.95% and *Prevotella oris* at 1.74%. It is worth mentioning the presence of *Fusobacterium nucleatum* at 2.33% belonging to the Fusobacteriota phylum (**Figures 1**, **2A, D**).

### Fecal microbiota

**Figure 3:**
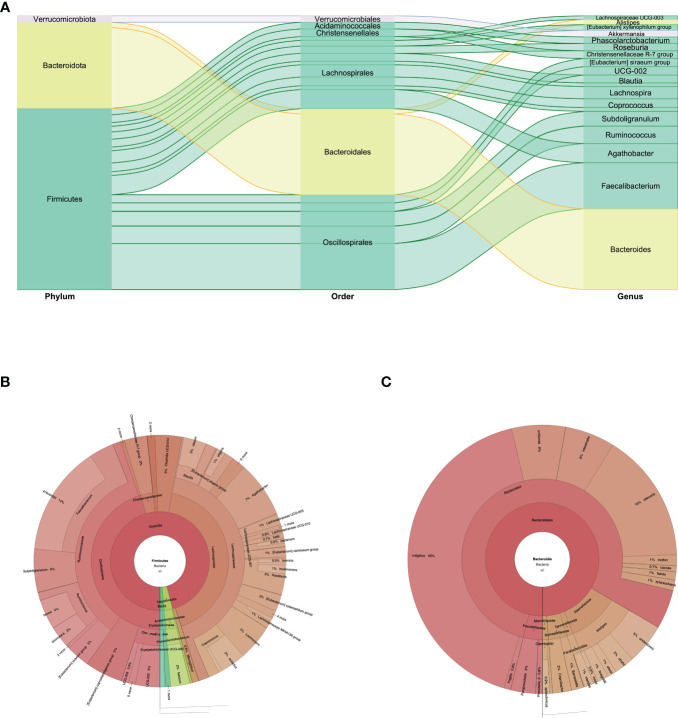
**(A)** Phylum, order, and genus enrichment in the fecal sample of the super-donor. Firmicutes (green), Bacteroidota (yellow), and Verrucomicrobiota (gray) were the most represented phyla in the fecal sample. **(B)** Taxonomic classification of the Firmicutes phylum. **(C)** Taxonomic classification of the Bacteroidota phylum.

In feces we identified 48 species. Firmicutes were the predominant phylum: two classes, Clostridia and Negativicutes, and four families for Clostridia; and the family with greater abundance was Ruminococcaceae, with the *Faecalibacterium* genus and the *Faecalibacterium prausnitzii* species at 9.87%. For the Lachnospiraceae family, the predominant genera were *Agathobacter* at 5% and *Lachnospira* at 3.2%. We found 2% of the Firmicutes phylum and Christensenellaceae family. In the Negativicutes class we found the Acidaminococcaceae family, with the *Phascolarctobacterium* genus and the *Phascolarctobacterium faecium* species at 1.7% (**Figures 1**, **3A, B**). The second phylum with greater abundance was Bacteroidota with two predominant families: Bacteroidaceae and Rikenellaceae. In the Bacteroidaceae and Bacteroides genus, the Bacteroides vulgatus specie predominated with an abundance of 11.8%, followed by Bacteroides stercoris at 4.4% abundance (**Figure 3C**). Regarding the Rikenellaceae family, the *Alistipes* genus, with the *Alistipes onderdonkii* species represented in 1.37% of all species found (**Figures 1**, **3A, B**). The third phylum in order of representation in the fecal sample was Verrucomicrobiota, Verrucomicrobiales order, Akkermansiaceae family, within which the *Akkermansia muciniphila* species presented an abundance of 1.7%. Other phyla present in a lesser amount were Proteobacteria and Actinobacteriota (**Figures 1**, **3A**).

## Discussion

Our study aimed to identify and describe the oral and fecal microbiota of a fecal matter super-donor for the treatment of dysbiosis presented in patients with diarrhea associated with *C. difficile* infection. This super-donor showed a wide diversity in oral and fecal microbiota, with a predominance of Firmicutes and Bacteroidota phyla, but with great variation in families, genera, and species depending on where they were found. We observed that only 2.2% of the microbiota, at the genus level, was similar in both the oral cavity and in the feces (**Supplementary 1**).

Overall, only 3% of potential candidates to donate product for FMTs are accepted, which shows how exceptional it is to find an ideal donor. Despite the advances in microbiota research, we still do not know what the properties of an “ideal” microbiota are (Kassam et al., 2019). We thus decided to explore the characteristics of our super-donor since all patients who received the product for FMT showed a favorable response to a single transplant, namely stopping the diarrhea.

The sequences obtained in the mouth were representative of the most common species, but were far from describing the total bacterial diversity (Arweiler and Netuschil, 2016). The donor in this study presented predominance in the Veillonellaceae family, with abundance at 27%, and the *Veillonella parvula* species at 19.7%. The *Veillonella parvula* species is of interest due to its presence in the supragingival and subgingival dental plaque of the human mouth, where it can cause opportunistic infections. In addition, the Veillonellaceae family is characterized by its mutualistic relationship with early, middle, and late colonizers of the mouth, becoming an important component in oral biofilm ecology (Gronow et al., 2010; Liu et al., 2011).

Another aspect that must be evaluated is the relationship between oral microbiota and other non-oral diseases. In this regard, the best example is the Fusobacteriota phylum, which includes species commonly found in the human oral cavity (*Fusobacterium* spp.) and in the intestinal tract (*Leptotrichia* spp.). Fusobacteria are divided into two families: Leptotrichiaceae and Fusobacteriaceae. The latter includes the *F. nucleatum* species, a multifaceted bacterium involved in various interactions and effects that are sometimes beneficial and sometimes harmful. *F. nucleatum* was for a long time described as being commonly found in oral microbiota, with integral and beneficial functions in the oral biofilm. However, it is considered an opportunistic pathogen that has the ability to maintain a symbiotic relationship with the host. Recently, a link between the presence of extraoral *F. nucleatum* and human colorectal cancer has been reported due to the presence of fusobacteria in tumor tissue (Castellarin et al., 2012; Kostic et al., 2012); however, the role of *F. nucleatum* as part of the cancer-causing microbiota is still being researched (Brennan and Garrett, 2019). It is important to define the causality of *F. nucleatum* and the diseases to which it has been associated, because this bacterium is ubiquitous in the mouth. However, its presence and abundance in the gut is related to inflammatory bowel disease and colorectal cancer. In our super-donor, we found *F. nucleatum* only in the mouth, not in feces.

The other family that predominated in this super-donor was Pasteurellaceae, which includes the *Haemophilus* genus, representing 22% of the oral microbiota with the *H. haemolyticus* and *H. quentini* species, and in a lesser amount *Haemophilus influenzae*, species that are dependent on factors X and V and are positive for urease and some for ornithine decarboxylase. These strains are considered rare and of little clinical significance (Nørskov-Lauritsen, 2014).

The third family that was highly present was Streptococcus, with *S. sanguinis* and *S. oralis* species predominating. These species are prominent in the mouth and are generally defined as commensals, but in immunocompromised subjects they are important opportunistic pathogens of systemic infections (Alves et al., 2019).

At the gut level, the composition of the microbiota is determined both by genetic and environmental influences and by diet, through a continuous process that can begin in the maternal uterus (Perez-Muñoz et al., 2017) and fluctuates throughout the life of each individual (Odamaki et al., 2016). In a healthy adult, the bacterial population in the intestine consists predominantly of members of the Firmicutes (60% to 80%) and Bacteroidetes (15% to 30%) (Human Microbiome Project Consortium, 2012) phyla, which are strictly anaerobic, and to a lesser degree Proteobacteria and Actinobacteria phyla. Therefore, our super-donor showed a diversity and distribution of the microbiota in normal feces similar to what is described in healthy subjects, with a predominance of the Firmicutes and Bacteroidota phyla, and a greater abundance of the *Bacteroides vulgatus* and *Faecalibacterium prausnitzii* species. The Human Microbiome Project established that *Bacteroides* are one of the most dominant genera in the healthy human gut and that they play an important role in keeping the gut ecosystem healthy (Human Microbiome Project Consortium, 2012). Patients with intestinal inflammation usually show lower *Bacteroides* levels (Conte et al., 2006; Zhou and Zhi, 2016). The *B. vulgatus* species reduces the inflammatory response in the gut, and has an immunoactive function involving the nucleotide-binding oligomerization domain 2 (NOD2) receptor. This receptor plays a critical role in the immune system by acting as a microbial sensor (Nigro et al., 2014). Ramanan et al. showed that *B. vulgatus* activates the immune response by translocation to the small intestine in a mouse model that lacked functionality in NOD2 (Ramanan et al., 2014). This is relevant because it indicates that the activity of species, such as *B. vulgatus*, may depend on the genetics of the host; it is also an endogenous modulator in the gut. The interaction of *B. vulgatus* with other bacteria, such as those of the genus *Akkermansia*, modulates the intestinal environment and, therefore, the health—or lack thereof—of the host (You et al., 2023). The abundance of *B. vulgatus* has a direct impact on the synthesis of microbial lipopolysaccharides in the human gut, with a protective effect against atherosclerosis (Yoshida et al., 2018).

The other most abundant bacterial species of human gut microbiota in healthy adults is *F. prausnitzii*—which in our super-donor accounted for 9.9% of the total bacterial population—as described in the literature (Miquel et al., 2013). This species is an anaerobic, commensal, metabolically active bacterium, and is considered the most important butyrate-producing bacterium in the human colon. To date, it has been considered as a bioindicator of human health since when its population decreases, it favors dysbiosis and other disorders such as inflammatory bowel disease, irritable bowel syndrome, and colorectal cancer, and has even been found in lower levels in dysbiosis due to acute COVID-19 disease, even after recovery (Ferreira-Halder et al., 2017; Baradaran Ghavami et al., 2021; Linares-Garcia et al., 2022). Researchers have also proposed that a decreased abundance of *F. prausnitzii* could be a predictive biomarker for *C. difficile* infection in patients with dysbiosis, because patients with this bacterial species have decreased numbers of butyrate-producing bacteria (*F. prausnitzii*) and increased the lactic acid-producing bacteria (*Akkermansia*). Even high levels of *Akkermansia muciniphila* can be a predictive marker for the onset of nosocomial diarrhea and worse prognosis in *C. difficile* infection. However, it has also been reported that *A. muciniphila* improves metabolic parameters, such as glucose homeostasis, and reduces inflammation in humans and mice (You et al., 2023). Our donor presented with a high abundance of *F. prausnitzii* and low abundance of *A. muciniphila*, similar to what has been described in healthy subjects (Vakili et al., 2020).

Feces donors who are rich in specific members of *Clostridium* groups IV and XIVa have also been reported to predict a sustained post-FMT response in patients with inflammatory bowel disease (Fuentes et al., 2017). The super-donor in our study also showed a predominance of *Clostridia* of the orders Lachnospirales and Oscillospirales, with greater abundance of Agathobacter and Lachnospira families. The genera of these families are known to hydrolyze starch and other sugars to produce butyrate and other short-chain fatty acids. All members of Lachnospiraceae are anaerobic, fermenters, and chemoorganotrophic, and some show strong hydrolyzing activity. For example, *Blautia* and *Roseburia* of the Lachnospiraceae family represent the genera most involved in the control of intestinal inflammatory processes, atherosclerosis, and maturation of the immune system, demonstrating that the final products of bacterial metabolism (butyrate) mediate these effects (Vacca et al., 2020). It has been reported that the gut microbiome of a super-donor is characterized by enrichment of Ruminococcaceae and Lachnospiraceae families, a characteristic that we observed in our super-donor (Moayyedi et al., 2015). Another interesting finding of this super-donor was the presence of Christsenellaceae, which represented 2% of the microbiotas in her stool. In fact, this family of the Clostridia class was described in 2012. It is widely found in America, Europe, Asia, Africa, and Australia; a greater relative abundance is observed in women, and it is associated with longevity. The relative abundance of Christensenellaceae in the human gut is inversely related to the BMI of the host, with low triglyceride levels, and elevated levels of high-density lipoproteins. In addition, Christensenellaceae is highly inheritable in multiple populations. Further research into the microbial ecology and metabolism of these bacteria should reveal the mechanical foundations of host–health associations, and enable their development as therapeutic agents, such as probiotics (Waters and Ley, 2019).

Because no two gut microbiomes are identical, the definition of a healthy gut microbiome remains undescribed. Despite this, having a stable and diverse gut community is generally correlated with a healthy intestinal state (Human Microbiome Project Consortium, 2012). The microbial diversity of the stool donor has been shown to be one of the most important factors influencing the FMT outcome. Vermeire et al. reported that donors whose products were effective in FMT have significantly greater bacterial richness than those who did not get a response (Vermeire et al., 2016).

The diversity of the microbiota of a super-donor, and the interaction between the different genera and species, plays a fundamental role in the effectiveness of its product in the FMT. This suggests that transplanted gut microorganisms have the ability to maintain or recover health, in a dynamic process between the microbiota and the host. Our findings encourage further research in order to develop bacterial therapies in infectious and inflammatory diseases.

## Data availability statement

The data presented in the study are deposited in the NCBI repository, accession number PRJNA974497.

## Ethics statement

The studies involving human participants were reviewed and approved by INCAN/CI/0170/18. The patients/participants provided their written informed consent to participate in this study. Written informed consent was obtained from the participant/patient(s) for the publication of this case report.

## Author contributions

Conception and design of study: NO-O, EF-F, and ER-G. Acquisition of data: EF-F, JA-D, and HM-O. Data analysis and/or interpretation: NO-O, EF-F, JA-D, and HM-O. Drafting of the manuscript and/or critical revision: NO-O, ER-G, and EF-F. Approval of the final version of the manuscript: NO-O, EF-F, JA-D, HM-O, and ER-G. All authors contributed to the article and approved the submitted version.
